# Gender-based differences in primary percutaneous coronary intervention in patients with myocardial infarction from a developing country: A retrospective cohort study

**DOI:** 10.1016/j.amsu.2022.103532

**Published:** 2022-04-01

**Authors:** Farah Yasmin, Sumeet Kumar, Manjeet Singh, Sagar, Karan Kumar, Om Parkash, Muhammad Sohaib Asghar, Fareeha Jawed, Tooba Ahmed Kirmani, Muhammad Tanveer Alam

**Affiliations:** a Department of Internal Medicine, Dow University of Health Sciences, Karachi, Pakistan; b Department of Internal Medicine, Ghulam Muhammad Mahar Medical College, Sukkur, Pakistan; c Department of Internal Medicine, Liaquat National Hospital and Medical College, Karachi, Pakistan; d Department of Internal Medicine, Jinnah Sindh Medical University, Karachi, Pakistan; e Department of Internal Medicine, Chandka Medical College, Larkana, Pakistan

**Keywords:** Gender disparity, Angiography, Outcomes, MACE, Adverse events

## Abstract

**Introduction:**

Limited data exists about gender's impact on differences in risk factors and outcomes in our setting. Therefore, we sought to ascertain sex-related differences in patients with AMI in our setting.

**Material and methods:**

This retrospective study analyzed data from 247 myocardial infarction patients hospitalized in a tertiary care hospital, between March and October 2020. After hospital admission, all patients underwent ECG, myocardial enzymes, troponin and other biochemical tests followed by primary PCI.

**Results:**

Patients were divided in two groups male (n = 153, mean age 55.2 ± 11.0 years) and female (n = 94, mean age 58.4 ± 12.7 years). The prevalence of smoking was higher in males than females (22.8% vs. 3.1%, p < 0.01) and so was history of three-vessel disease (3VD; 18.9% vs. 7.4%, p = 0.013). History of myocardial infarction was lower in females than males (13.8% vs. 24.8%, p = 0.03) however the age did not vary significantly between the two groups (p = 0.21). Serum creatinine (sCr) levels (1.0 ± 0.77 μmol/L vs. 1.2 ± 0.73 μmol/L, p = 0.28) and body mass index (28.4 ± 5.3 vs 27.4 ± 4.8, p = 0.45) were lower in females as compared to males, however not statistically significant. The incidence of major adverse events, severe arrhythmia and in-hospital outcomes showed no significant difference (p > 0.05) between the two groups. Post-op TIMI score and average length of hospital stay were not statistically different either (3.29 ± 2.9 vs. 2.6 ± 1.7, p = 0.726).

**Conclusion:**

Our study shows that females have a comparable age of onset of major cardiovascular events as that of males. Post-PCI clinical outcomes and in-hospital stay had no significant differences between the two groups.

## Highlights

What is already known on this topic:

• Limited data exists about gender's impact on differences in risk factors and outcomes STEMI in developing countries like Pakistan.
• Previous analyses on gender disparities have revealed higher mortality in females compared to males when hospitalized for STEMI.
What this study adds:
• The incidence of major adverse events during in-hospital stay showed no significant difference among gender.
• The prevalence of smoking and 3VD in males was higher than in females. Despite of differences in co-morbidities between the groups, clinical outcomes remained consistent.

## 1 Introduction

The influence of patient gender on outcomes of acute myocardial infarction (AMI) is controversial. Previous analyses on gender disparities have revealed higher mortality in females compared to males when hospitalized for STEMI [1]. This can be due to differences in presentation, age of AMI, risk factors and patient and system related delays [2]. Limited data exists about gender's impact on differences in risk factors and outcomes in our setting. Therefore, we attempted to analyze the clinical course, characteristics and outcomes of patient on gender-based differences presented with myocardial infarction going under primary percutaneous coronary intervention.

## 2 Material and methods

This retrospective study analyzed data from 247 myocardial infarction patients hospitalized in a tertiary care hospital, between March and October 2020. The study has been reported in line with the STROCSS criteria 2021 [3]. The research was registered with registry of Dow University Hospital with UIN # IRB/DUH/2020/784. The diagnostic criteria for ACS were based on chest pain or discomfort, ECG, and measurements of myocardial injury biomarkers. After hospital admission, all patients underwent ECG, myocardial enzymes, troponin and other biochemical tests followed by primary PCI. Hospital discharge (or death without discharge) was primary end outcome. Data was gathered for Socio-demographic and clinical variables, Angiographic profile, and In-hospital outcomes from retrospective chart review of all the patients. Clinical variables included comorbidities, history of previous cardiac events, presenting symptoms, Killip class, and infarction localization on ECG. Angiographic profile included vessel involvement, post-operative TIMI score, severity of lesion on angiography, and number of stents placed. In-hospital outcomes reported length of hospital stay, exacerbation of CHF during hospitalisation, any bleeding complication, arrhythmia, new onset MI, stroke, cardiogenic shock, and mortality. Data was analyzed using SPSS version 25.0, and descriptive statistics were used to report frequencies while Chi-square/Independent T-tests were utilized to determine the differences in variables between males and females.

## 3 Results

Patients were divided in two groups male (n = 153, mean age 55.2 ± 11.0 years) and female (n = 94, mean age 58.4 ± 12.7 years). Mean BMI was 27.78 ± 5.01 kg/m^2^. Prior history of MI was present in 20.64% of the participants while Prior history of CABG in 4.04%. Frequently reported comorbidities were hypertension (70.04%), diabetes (46.96%), renal insufficiency (11.33%), atrial fibrillation (5.26%), and stroke (4.04%).

The prevalence of smoking was higher in males than females (22.8% vs. 3.1%, p < 0.01) and so was history of three-vessel disease (3VD; 18.9% vs. 7.4%, p = 0.013). History of myocardial infarction was lower in females than males (13.8% vs. 24.8%, p = 0.03), however the age did not vary significantly between the two groups (p = 0.21). Serum creatinine (sCr) levels (1.0 ± 0.77 μmol/L vs. 1.2 ± 0.73 μmol/L, p = 0.28) and body mass index (28.4 ± 5.3 vs 27.4 ± 4.8, p = 0.45) were lower in females as compared to males, however not statistically significant as shown in Table 1.

**Table 1:** Sociodemographic and clinical data of the study population.

Socio-demographic and clinical variables	**Males (n** = **153)**	**Females (n** = **94)**	**P-value**
**Age (years)**	55.2 ± 11.0	58.4 ± 12.7	0.21
**BMI**	27.4 ± 4.8	28.4 ± 5.3	0.45
Smoker	35 (22.8%)	3 (3.1%)	<0.01
Non-smoker	118 (77.1%)	91 (96.8%)	<0.01
Peripheral artery disease (%)	3 (1.96%)	2 (2.1%)	0.93
Dyslipidemia (%)	38 (24.8%)	19 (20.2%)	0.402
Hypertension (%)	100 (65.3%)	73 (77.6%)	0.040
**Diabetes mellitus (%)**	71 (46.4%)	45 (47.8%)	0.823
**Atrial fibrillation (%)**	7 (4.5%)	6 (6.4%)	0.537
**History of Stroke (%)**	7 (4.5%)	3 (3.2%)	0.592
**Renal insufficiency (%)**	18 (11.7%)	10 (10.6%)	0.786
**On hemodialysis (%)**	6 (3.9%)	3 (3.2%)	0.766
**History of cardiac arrest (%)**	4 (2.6%)	0 (0%)	0.114
Prior MI (%)	38 (24.8%)	13 (13.8%)	0.038
**Prior PCI (%)**	32 (20.9%)	19 (20.2%)	0.895
**Prior CABG (%)**	9 (5.8%)	1 (1%)	0.062
**Presenting symptoms** **Typical angina chest pain (%)** **Atypical angina chest pain (%)** **No chest pain (%)**	78(50.9%) 19 (12.4%) 52 (33.9%)	44 (46.8%) 14 (14.8%) 32 (34%)	0.524 0.579 0.993
**Killip class (preoperative, %)** **1** **2** **3** **4**	66 (43.1%) 74 (48.3%) 8 (5.2%) 5 (3.2%)	38 (40.4%) 46 (48.9%) 5 (5.3%) 5 (5.3%)	0.726 0.931 0.975 0.427
**MI localization in ECG (%)** **Anterior wall** **Inferior wall** **Anterolateral wall** **Others**	10 (6.5%) 10 (6.5%) 3 (1.9%) 1 (0.6%)	7 (7.4%) 3 (3.2%) 1 (1%) 4 (4.2%)	0.784 0.253 0.588 0.051
**Troponin I on admission (pg/ml)**	1743 ± 6417	1474 ± 5719	0.309
**sCR on admission (umol/L)**	1.2 ± 0.73	1.0 ± 0.77	0.288
**LV ejection fraction** ≤ **40%:**	25 (16.3%)	17 (18%)	0.935

History of hypertension was significant in females (p = 0.04), while rest comorbidities showed no difference. Right coronary artery was significantly more likely to be infarct–related artery among males (p = 0.021). Post-op TIMI score (p = 0.247) and average length of hospital stay were not statistically different either (3.29 ± 2.9 vs. 2.6 ± 1.7, p = 0.726) as shown in Table 2. The incidence of major adverse events, severe arrhythmia and in-hospital outcomes showed no significant difference (p > 0.05) between the two groups as shown in Table 3.

**Table 2:** Angiographic profile of the study population.

Angiographic profile	Males (n = 153)	Females (n = 94)	P-value
Infarct-related coronary artery (%) Left main coronary artery Left anterior descending Circumflex artery Right coronary artery	6 (3.9%) 45 (29.4%)26 (16.9%) 36 (23.5%)	1 (1%) 23 (24%) 12 (12.7%) 11 (11.7%)	0.189 0.398 0.371 0.021
Emergency call (%)	5 (3.2%)	5 (5.3%)	0.427
Severity of CAD (%) One-vessel: Two-vessel: Three-vessel:	30 (19.6%) 13 (8.5%) 29 (18.9%)	16 (17%) 8 (8.5%) 7 (7.4%)	0.612 0.997 0.013
Post-operative TIMI (%) 3 2 0–1	104 (67.9%) 33 (21.5%) 16 (10.4%)	54 (57.4%) 27 (28.7%) 13 (13.8%)	0.247
Medical therapy (%) Aspirin Clopidogrel/Tegreino β-blocks ACEI/ARB Statins	126 (82.3%) 75 (49%) 100 (65.3%) 76 (49.6%) 120 (78.4%)	69 (73.4%) 47 (50%) 70 (74.4%) 36 (38.2%) 74 (78.7%)	0.094 0.881 0.133 0.081 0.957
Successful PCI (%)	138 (90%)	81 (86%)	0.333
Stent number (Number of implanted stents)	2.6 ± 0.968	2.7 ± 1.1	0.687
Average length of hospital stays (days)	3.29 ± 2.9	2.6 ± 1.7	0.726

**Table 3:** In-hospital outcomes observed during hospital stay.

In-hospital outcomes	Males (n = 153)	Females (n = 94)	P-value
CHF need treatment	14 (9%)	10 (10.6%)	0.701
Bleeding complication	4 (2.6%)	1 (1%)	0.401
Severe arrhythmia	2 (1.3%)	2 (2%)	0.620
Myocardial infarction	4 (2.6%)	3 (3.2%)	0.791
Stroke	0 (0%)	1 (1%)	0.201
Cardiogenic shock	5 (3.2%)	1 (1%)	0.275
In-hospital death	4 (2.6%)	4 (4.2%)	0.479

**Fig. 1:**
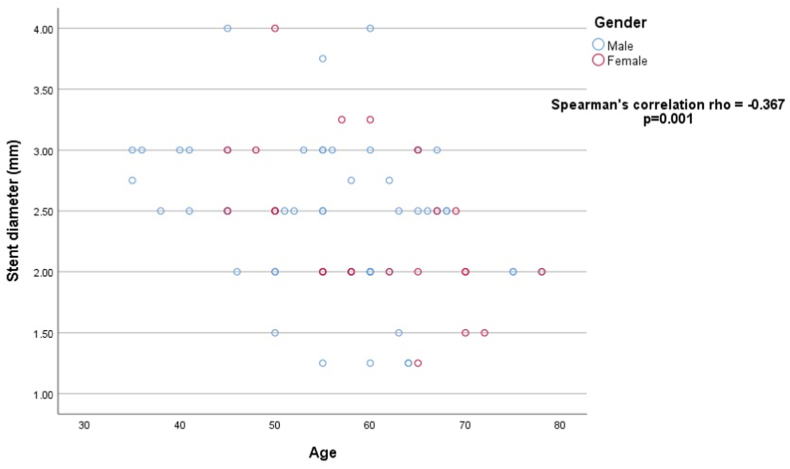
Scatter plot showing correlation of stent diameter (mm) with age of the study participant.

**Fig. 2:**
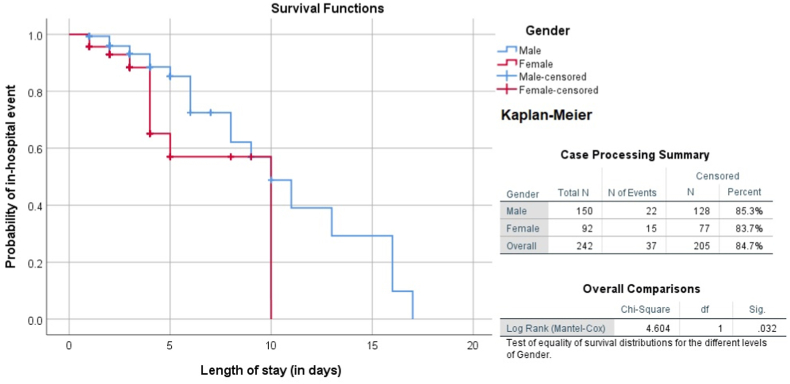
Kaplan-Meier survival curve showing relation of in-hospital outcomes with gender.

Stent diameter (mm) had a negative correlation with age i.e., with increasing age stent diameter tends to be decreased in both gender (p = 0.001) as shown in Fig. 1. Furthermore, females showed a stronger correlation than males. On Kaplan-Meier analysis, major in-hospital events occurred early in females than males during hospital stay post-PCI (log rank p = 0.032, chi-square 4.604) with hazard ratio of 2.105 [95% confidence interval: 1.032–4.294] p = 0.041 as shown in Fig. 2.

## 4 Discussion

One finding of the study was a difference between comorbidities at baseline but no significant difference in clinical outcome between two groups. However previous studies showed that women had poor outcome than men, once PCI is done [4,5]. The studies conducted in China, Sweden and Belgium showed that women presented at an older age than men with AMI [[6], [7], [8]]. This is likely due to protective effects of oestrogen that helps women to combat coronary atherosclerosis until menopause and could potentially be offset by the undiagnosed/untreated medical conditions that are directly linked to coronary artery disease.

When it comes to comorbidities, we found out around 77.6% of women were hypertensive as opposed to 65.3% of men at time of presentation at hospital with ACS. One study conducted in America also showed that women are increasingly hypertensive than men with ACS [9]. This may be due to the limited access to healthcare for the women. It might be difficult to predispose it to certain socio-demographic factors since similar factors would have an impact of the rates of diabetes mellitus as well. But, in case of diabetes, 47.8% of women vs 46.4% of men were diabetic. Another study showed that women at time of presentation are presumably diabetic in greater number than men [10]. Men (22.8%) were smoker in greater number than women (3.1%) according to our research. In literature many studies show the evidence that history of smoking is not common in women [11,12].

At the time of coronary angiography, three vessel disease was significantly higher in males than females. Many studies in past showed that females are less likely to have multi-vessel disease or severe CAD than their male counterpart [8,13]. Moreover, our study showed that only 1% of women had left main artery disease as opposed to 3.9% of men with similar findings. While group of researchers in Malaysia observed that left main artery was affected more in women than men [14].

In our study there were 9% men who needed in-hospital treatment for CHF in contrast with 10.6% women (p-value 0.701). However, there are many studies done in past, that tell women are more likely to have heart failure than men during hospitalisation [6,12,15,16]. On addressing bleeding complications, our data revealed that 2.6% men in comparison with only 1% of women faced these complications. While one study published in American Journal of Cardiology suggests that women are more likely to have bleeding than men, unexplained by all factors taken into consideration like differences in comorbidities, angiographic profile and medical therapy [4]. In literature previously it had been found out that women have higher heart rate in resting state than men, possibly due to variation in intrinsic properties of sinus node, autonomic nervous system involvement and exercise tolerance [10,17]. In our study, only small number of both men and women had severe arrhythmia during hospitalisation and no remarkable gender-based difference was observed. One study done in Finland showed that females have less chances of subsequent MI than men [18]. Moreover, our study showed that 2.6% men vs 3.2% women had subsequent new-onset MI. A study done in England also concluded that both genders do not have remarkable difference in rate of stroke [12]. There are number of researches in literature that suggest women have higher in hospital mortality when compared with men. But we found out that there is no significant difference between two genders when it comes to in-hospital death [11,14,19].

Therefore, we conclude that women have comparable age of onset of cardiovascular events, both genders differ in comorbidities at time of presentation with AMI. But there are no significant differences witnessed in case of in-hospital outcome. Certain limitations affect the generalizability of current findings as there are various factors like access to healthcare and traditional societal values may have an impact on the perceived differences and the disease prevalence could potentially be more comparable. There could also be an element of detection bias as men with pre-existing diagnosed CAD are more likely to receive treatment than in women who have undiagnosed disease.

## Ethical approval

Ethical approval was taken in this study from institutional review board of Dow University Hospital (Ref:App.# IRB/DUH/2020/784).

## Source of funding

The authors declare that they have no commercial associations (e.g. consultancies, stock ownership, equity interest, patent/licensing arrangement etc.) or funding with this article.

## Author contribution

M.T.A, M.S.A, and F.Y conceived the idea, S.K, M.S, K.K, and O.P retrieved the data, did write up of letter, and finally T.A.K, F.J, and S, reviewed and provided inputs. All authors approved the final version of the manuscript.

## Trial registry number

1. Name of the registry: Dow University Hospital.
2. Unique Identifying number or registration ID: IRB/DUH/2020/784
3. Hyperlink to your specific registration (must be publicly accessible and will be checked):

## Guarantor

Muhammad Sohaib Asghar.

## Patient consent

Consent to participate from the patients was waived and not required due to retrospective nature of the data collection.

## Provenance and peer review

Not commissioned, externally peer reviewed.

## Declaration of competing interest

The authors have no conflict of interest.

## Data Availability

Data can be made available on request from corresponding author.
